# Association of intraoperative hyperglycemia and postoperative outcomes in patients undergoing non-cardiac surgery: a multicenter retrospective study

**DOI:** 10.1186/s12871-020-01022-w

**Published:** 2020-05-07

**Authors:** Nirav J. Shah, Aleda Leis, Sachin Kheterpal, Michael J. Englesbe, Sathish S. Kumar

**Affiliations:** 1 Department of Anesthesiology, University of Michigan Medical School, H247 UH, SPC 5048, 1500 East Medical Center Drive, Ann Arbor, MI 48109-5048 USA; 2 Department of Surgery, University of Michigan Medical School, Ann Arbor, MI USA

**Keywords:** Hyperglycemia, Complications, Anesthesiology

## Abstract

**Background:**

While pre and postoperative hyperglycemia is associated with increased risk of surgical site infection, myocardial infarction, stroke and risk of death, there are no multicenter data regarding the association of intraoperative blood glucose levels and outcomes for the non-cardiac surgical population.

**Methods:**

We conducted a retrospective cohort study from the Michigan Surgical Quality Collaborative, a network of 64 hospitals that prospectively collects validated data on surgical patients for the purpose of quality improvement. We included data for adult general, vascular, endocrine, hepatobiliary, and gastrointestinal operations between 2013 and 2015. We assessed the risk-adjusted, independent relationship between intraoperative hyperglycemia (glucose > 180) and the primary outcome of 30-day morbidity/mortality and secondary outcome of infectious complications using multivariable logistic regression modelling. Post hoc sensitivity analysis to assess the association between blood glucose values ≥250 mg/dL and outcomes was also performed.

**Results:**

Ninety-two thousand seven hundred fifty-one patients underwent surgery between 2013 and 2015 and 5014 (5.4%) had glucose testing intra-operatively. Of these patients, 1647 patients (32.9%) experienced the primary outcome, and 909 (18.1%) the secondary outcome. After controlling for patient comorbidities and surgical factors, peak intraoperative glucose > 180 mg/dL was not an independent predictor of 30-day mortality/morbidity (adjusted OR 1.05, 95%CI:0.86 to 1.28; *p*-value 0.623; model c-statistic of 0.720) or 30-day infectious complications (adjusted OR 0.93, 95%CI:0.74,1.16; p 0.502; model c-statistic of 0.709). Subgroup analysis for patients with or without diabetes yielded similar results. Sensitivity analysis demonstrated blood glucose of 250 mg/dL was a predictor of 30-day mortality/morbidity (adjusted OR: 1.59, 95% CI: 1.24, 2.05; *p* < 0.001).

**Conclusions:**

Among more than 5000 patients across 64 hospitals who had glucose measurements during surgery, there was no difference in postoperative outcomes between patients who had intraoperative glucose > 180 mg/ dL compared to patients with glucose values ≤180 mg/ dL.

## Background

Patients undergoing surgery may have high glucose values, regardless of whether they have diabetes. Perioperative hyperglycemia has been shown to be associated with increased risk of surgical site infection, myocardial infarction, stroke and risk of death [1–3]. Stress hyperglycemia (hyperglycemia without diagnosis of diabetes) can develop with surgery and critical illness, and is more common during cardiac surgery. Evidence suggests that outcomes for patients with stress hyperglycemia are worse than in patients with hyperglycemia who have diabetes [4–7].

Most of the published literature related to outcomes in patients with intraoperative hyperglycemia has been in the cardiac surgical population, and there is growing evidence on the appropriate treatment of perioperative high glucose levels in this group [8]. Blaha et al. found that adhering to a tight glucose control protocol starting in the intraoperative period, instead of postoperatively, reduced perioperative adverse events, especially for non-diabetics [9].

Other studies have demonstrated associations between perioperative hyperglycemia and post-operative morbidity in the noncardiac surgical population, using data obtained pre- and post-operatively, but these studies do not take intra-operative values into account [10–12]. Despite evidence that high glucose levels need to be addressed in the perioperative setting, there is little data focusing on intraoperative glucose levels and outcomes for the noncardiac surgical population [10]. Small, single center analyses have demonstrated a tenuous relationship between severe hyperglycemia and postoperative infectious complications; however, these data suffer from overfit models or data from more than decade ago [13]. As a result, the association between intraoperative glucose and outcomes remain controversial. While most anesthesiologists would acknowledge that treatment of “high” glucose during surgery may improve postoperative outcomes, they may also worry that symptoms of hypoglycemia are masked by general anesthesia, posing a unique risk to aggressive glycemic management during the intraoperative period. Additionally, there are workflow factors that limit compliance with this important intervention, such as access to point of care glucose measuring devices. However, the use of real-time alerting systems has been shown to modify glucose-checking behavior and improve compliance [14, 15].

The aim of our study was to elucidate the relationship between intraoperative hyperglycemia and postoperative outcomes using a large multicenter registry reflecting small and large community hospitals and academic centers, with a variety of care processes and patient profiles. We hypothesized that intraoperative hyperglycemia (peak glucose > 180 mg/dL or 10 mmol/L between the time points anesthesia start and anesthesia end) during noncardiac surgery is an independent predictor of combined 30-day morbidity and mortality after controlling for known patient and procedural risk factors.

## Methods

We conducted a retrospective cohort study from the Michigan Surgical Quality Collaborative (MSQC), a voluntary network of approximately 70 hospitals that collects data on surgical patients for the purpose of quality improvement and research using a foundation of the National Surgical Quality Improvement Program data elements and methodology [16, 17]. The MSQC is funded by Blue Cross Blue Shield of Michigan, a private, not-for-profit insurance company. Although Blue Cross Blue Shield provides financial support for the project, they are not involved in the policy recommendations that are developed within the collaborative. MSQC hospitals are predominantly community hospitals but do include several teaching facilities with surgical and/or medical residents. Patient selection uses an algorithm designed to minimize selection bias. Cases are reviewed using a sampling algorithm designed to minimize selection bias and represent 90% of eligible cases, approximately 50,000 cases per year [18]. De-identified MSQC data collection for quality improvement is Institutional Review Board exempt; the current study using a limited data set derived from the MSQC database was approved by the University of Michigan Institutional Review Board review (HUM 00091060).

We included data for adult general, vascular, endocrine, hepatobiliary and gastrointestinal (upper and colorectal) cases between 2013 and 2015 and excluded patients with American Society of Anesthesia Classification (ASA) 5 or 6. Each participating hospital employs at least one trained Surgical Clinical Quality Reviewer to prospectively collect data on surgery patients, their operations, and 30-day outcomes. Patient data collected from the electronic or paper medical record included demographics (age, gender, body mass index (BMI), ASA class, emergent status, surgical procedure group), preoperative comorbidities (diabetes, ventilator dependence, chronic obstructive pulmonary disease (COPD), pneumonia, ascites, congestive heart failure, hypertension, history of peripheral vascular disease, currently requiring or on dialysis, disseminated cancer, open wound, use of steroids/immunosuppressive medications for chronic condition, > 10% loss of body weight in the 6 months prior to surgery, alcohol use > 2 drinks/day in the 2 weeks prior to surgery, presence of sleep apnea, cigarette use within 1 year, presence of sepsis or severe sepsis within 48 h prior to surgery, history of coronary artery disease, and history of deep vein thrombosis), intraoperative characteristics (surgical time, peak blood glucose measurements, insulin administration), and postoperative outcomes (Appendix A). Although the definition of intraoperative hyperglycemia remains controversial, a specific threshold is necessary for a robust, pre-planned primary analysis. We selected a glucose of 180 mg/dL given that several studies have shown an association between inpatient hyperglycemia (defined as greater than 180 mg/dL) and adverse clinical outcomes [10, 12]. This manuscript was drafted adherent to the applicable STROBE guidelines [19].

### Outcomes

The primary outcome was combined 30-day mortality / morbidity including infectious, cardiovascular, thromboembolic, and neurologic adverse events as detailed in Appendix A. The secondary outcome was 30-day infectious complications including surgical site infections, pneumonia, urinary tract infections, sepsis, central line associated bloodstream infections, and *Clostridium difficile* infection. Each of these complications was prospectively collected by a trained nurse data collector per MSQC definitions and processes [16].

### Statistical analysis

Univariate associations were used to compare demographic and clinical characteristics among patients with a peak glucose > 180 mg/dL to those with glucose ≤180 mg/dL, and also with and without history of diabetes in the entire patient cohort and in the cohort of patients who underwent glucose testing (cohort study group). Normality of all continuous data was checked using the Kolmogorov-Smirnov test. Data are presented as frequencies with percentages for categorical variables and medians with 25th and 75th percentiles for continuous variables. Univariate differences were assessed using Chi-square or Fisher’s Exact tests for categorical variables and Mann-Whitney U or Kruskal-Wallis tests for continuous variables, as appropriate.

Non-parsimonious multivariable logistic regression models were used for the primary and secondary outcomes to determine if glucose > 180 mg/dL was an independent predictor of the primary or secondary outcomes. Variables chosen for model inclusion based on clinical significance were: age, gender, race, World Health Organization Body Mass Index classification, ASA class, procedure, urgent/emergent case status, year of case, intraoperative administration of insulin, surgical duration, intraoperative blood glucose > 180 mg/dL, and total number of comorbidities (diabetes, ventilator dependence, COPD, pneumonia, ascites, congestive heart failure, hypertension, history of peripheral vascular disease, currently requiring or on dialysis, disseminated cancer, open wound, use of steroids/immunosuppressive medications for chronic condition, > 10% loss of body weight in the 6 months prior to surgery, alcohol use > 2 drinks/day in the 2 weeks prior to surgery, presence of sleep apnea, cigarette use within 1 year, presence of sepsis or severe sepsis within 48 h prior to surgery, history of coronary artery disease, and history of deep vein thrombosis). Before any models were constructed, covariates were assessed for collinearity using a Pearson’s correlation matrix. Pairs of variables with a correlation > 0.70 were deemed to be collinear, and the variable with the larger univariate effect size was kept in the model. All other variables were entered into the model. Any covariate deemed to be statistically significant in the model after adjusting for all other variables was considered to be an independent predictor of the outcome.

We performed a pre-planned sensitivity analysis to assess the impact of a tight glucose threshold by using glucose > 150 mg/dL as the independent predictor with the same multivariable logistic regression model. A glucose of 150 mg/dL was used for the sensitivity analysis as several previous studies have used this threshold to define strict control [20, 21]. In addition, we performed the following pre-planned subgroup analyses: elective cases, non-diabetic cases, inpatient/admit patients, and surgical duration greater than or equal to 60 min with glucose > 180 mg/dL as the independent predictor. A post hoc sensitivity analysis to assess the association between blood glucose values ≥250 mg/dL and outcomes was performed in response to reviewer requests. If missing, surgical times were imputed as the median time (represented by the other cases in the database) for the primary surgical CPT. Missing BMI were also imputed.

A *p*-value of < 0.05 was considered statistically significant for all analyses. Measures of effect size for all logistic regression models were reported as adjusted odds ratio and 95% confidence intervals for all model covariates. All analysis was conducted using SAS version 9.4 (SAS Institute, Cary, NC) and SPSS version 24 (IBM).

## Results

**Fig. 1:**
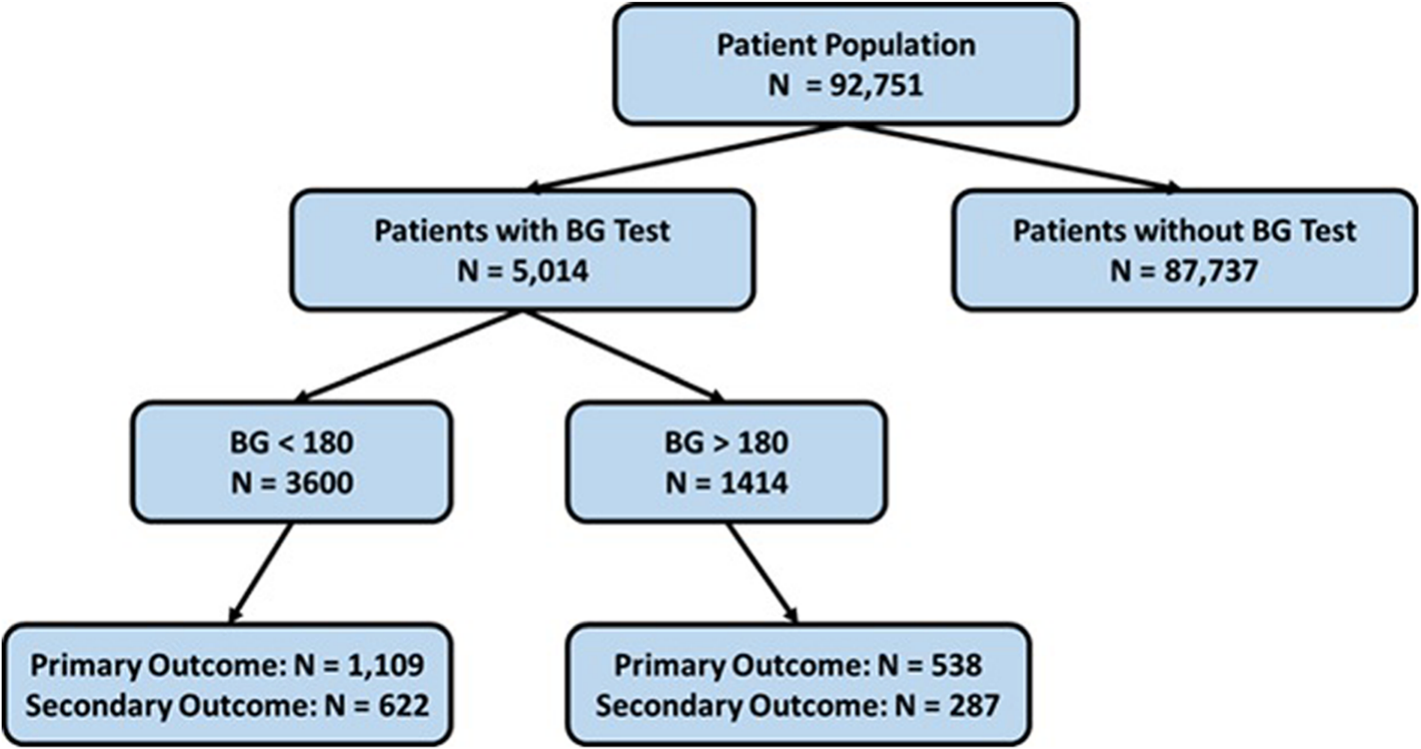
Patient Population Flowchart

Of the 92,751 patients who underwent general, hepatobiliary, gastrointestinal (GI), vascular, and endocrine surgery from 2013 to 2015, the study cohort consisted of 5014 patients (5.4%) who had intraoperative glucose testing performed (Fig. 1). Patients with blood glucose testing had significantly more comorbidities (except for alcohol and tobacco use), were older, had longer surgeries, and worse outcomes than those who did not receive glucose testing (Table 1). In the full study population, 18,191 out of 92,751 patients (19.6%) had a history of diabetes.

**Table 1:** Demographics and clinical characteristics for full patient population

	Blood Glucose Recorded Intraop (*N* = 5014) n(%)	Blood Glucose Not Recorded Intraop (*N* = 87,737) n(%)	*P*-value
**Patient Demographics**			
Age	66.0 [57.0 to 74.0]	56.0 [42.0 to 68.0]	< 0.001
*WHO BMI Classification*			< 0.001
Underweight	102 (2.0)	1717 (2.0)	
Normal	1056 (21.1)	20,849 (23.8)	
Overweight	1429 (28.5)	26,636 (30.4)	
Obese	2427 (48.4)	38,535 (43.9)	
ASA Class 3 or 4	4449 (88.7)	42,175 (48.1)	< 0.001
Urgent/Emergent Case	1874 (37.4)	35,180 (40.1)	< 0.001
*Surgical Procedure Group*			< 0.001
General	740 (14.8)	19,585 (22.3)	
Endocrine	410 (8.2)	4776 (5.4)	
Hepatobiliary	560 (11.2)	25,816 (29.4)	
GI (Upper and Colorectal)	1640 (32.7)	28,374 (32.3)	
Vascular	1664 (33.2)	9186 (10.5)	
**Pre-operative Clinical Characteristics**			
*Pre-op Sepsis*			< 0.001
None	4560 (91.0)	81,197 (92.6)	
Sepsis	232 (4.6)	4944 (5.6)	
Severe Sepsis	222 (4.4)	1596 (1.8)	
Diabetes	3231 (64.4)	14,976 (17.1)	< 0.001
Sleep Apnea	1061 (21.2)	10,576 (12.1)	< 0.001
Cancer	212 (4.2)	1199 (1.4)	< 0.001
CHF	112 (2.2)	706 (0.8)	< 0.001
COPD	790 (15.8)	8001 (9.1)	< 0.001
CAD	1722 (34.3)	13,725 (15.6)	< 0.001
DVT/PE^a^	542 (11.3)	4656 (5.8)	< 0.001
ETOH > 2 drinks/day in the 2 weeks prior to admission	145 (2.9)	2727 (3.1)	0.390
Tobacco use within 1 year - cigarette	1367 (27.3)	23,091 (26.3)	0.140
Hypertension	3831 (76.4)	41,833 (47.7)	< 0.001
Pneumonia	80 (1.6)	493 (0.6)	< 0.001
HbA1c^a^	7.2 [6.3 to 8.7]	6.1 [5.6 to 7.2]	< 0.001
Creatinine^a^	0.9 [0.7 to 1.3]	0.8 [0.7 to 1.0]	< 0.001
Ascites	98 (2.0)	736 (0.8)	< 0.001
10% Loss of Body Weight 6 Months Before Surgery	203 (4.1)	1409 (1.6)	< 0.001
Steroids/Immunosuppressive Meds for chronic condition	304 (6.1)	2973 (3.4)	< 0.001
Currently Requires or is on Dialysis	223 (4.5)	1170 (1.3)	< 0.001
Open Wound With or Without Infection	657 (13.1)	3637 (4.2)	< 0.001
Peripheral Vascular Disease	1169 (23.3)	7119 (8.1)	< 0.001
Ventilator Dependent	96 (1.9)	374 (0.4)	< 0.001
**Intraoperative Characteristics**			
Surgical Time (Minutes)	157.0 [98.0 to 251.0]	72.0 [45.0 to 116.0]	< 0.001
**Outcomes**			
30 Day Morbidity and Mortality^a^	1647 (32.9)	10,341 (11.8)	< 0.001
Infectious Complications	909 (18.1)	5874 (6.7)	< 0.001

Data are presented as frequency (%) or median [25th percentile to 75th percentile], as appropriate

^a^Percentages are given as percent of the non-missing number of values in that group

Of the glucose testing cohort, 1647 patients (32.9%) experienced the primary outcome of 30-day morbidity/mortality, and 909 (18.1%) the secondary outcome of 30-day infectious complications. Of the glucose testing cohort, 1414 patients (28.2%) had a glucose > 180 mg/dL (Table 2). These patients were more likely to have diabetes (76.4% vs. 59.8%, *p* < 0.001), hypertension (79.4% vs. 75.2%, *p* = 0.002), obesity (56.1% vs. 45.7%, p < 0.001), and intraoperative insulin administration (55.7% vs. 6.6%, p < 0.001). Those with a glucose > 180 mg/dL were less likely to have coronary artery diease (CAD) (32.0% vs. 35.3%, *p* = 0.026) and were of slightly younger age (median 65.0 vs. 66.0, *p* = 0.003). Unadjusted infectious complication rates and 30-day morbidity and mortality rates were significantly higher in the glucose > 180 mg/dL group (20.3% vs. 17.3%, *p* = 0.013; 38.1% vs. 30.9%, *p* < 0.001).

**Table 2:** Univariate comparison of demographics and clinical characteristics for study cohort

	Blood Glucose <= 180 (*N* = 3600) n(%)	Blood Glucose > 180 (*N* = 1414) n(%)	*P*-value
**Patient Demographics**			
Age	66.0 [57.0 to 74.0]	65.0 [56.0 to 72.0]	0.003
*WHO BMI Classification*			< 0.001
Underweight	75 (2.1)	23 (1.6)	
Normal	818 (22.7)	232 (16.4)	
Overweight	1063 (29.5)	366 (25.9)	
Obese	1644 (45.7)	793 (56.1)	
Female Gender	1628 (45.2)	677 (47.9)	0.090
ASA Class 3 or 4	3184 (88.4)	1265 (89.5)	0.305
Urgent/Emergent Case	1334 (37.1)	540 (38.2)	0.455
*Surgical Procedure Group*			0.011
General	539 (15.0)	201 (14.2)	
Endocrine	279 (7.8)	131 (9.3)	
Hepatobiliary	382 (10.6)	178 (12.6)	
GI (Upper and Colorectal)	1162 (32.3)	478 (33.8)	
Vascular	1238 (34.4)	426 (30.1)	
**Pre-operative Clinical Characteristics**			
*Pre-op Sepsis*			0.005
None	3293 (91.5)	1267 (89.6)	
Sepsis	145 (4.0)	87 (6.2)	
Severe Sepsis	162 (4.5)	60 (4.2)	
Diabetes	2151 (59.8)	1080 (76.4)	< 0.001
Sleep Apnea	729 (20.3)	332 (23.5)	0.012
Cancer	156 (4.3)	56 (4.0)	0.555
CHF	87 (2.4)	25 (1.8)	0.162
COPD	574 (15.9)	216 (15.3)	0.559
CAD	1270 (35.3)	452 (32.0)	0.026
DVT/PE^a^	392 (11.4)	150 (11.0)	0.711
ETOH > 2 drinks/day in the 2 weeks prior to admission	113 (3.1)	32 (2.3)	0.096
Tobacco use within 1 year - cigarette	1011 (28.1)	356 (25.2)	0.038
Hypertension	2708 (75.2)	1123 (79.4)	0.002
Pneumonia	53 (1.5)	27 (1.9)	0.266
HbA1c^a^	6.9 [6.1 to 8.2]	8.0 [6.9 to 9.6]	< 0.001
Creatinine^a^	0.9 [0.7 to 1.2]	0.9 [0.7 to 1.3]	0.313
Ascites	77 (2.1)	21 (1.5)	0.132
10% Loss of Body Weight 6 Months Before Surgery	148 (4.1)	55 (3.9)	0.720
Steroids/Immunosuppressive Meds for chronic condition	220 (6.1)	84 (5.9)	0.820
Currently Requires or is on Dialysis	184 (5.1)	39 (2.8)	< 0.001
Open Wound With or Without Infection	502 (13.9)	155 (11.0)	0.005
Peripheral Vascular Disease	839 (23.3)	330 (23.3)	0.981
Ventilator Dependent	67 (1.9)	29 (2.1)	0.659
**Intraoperative Characteristics**			
Surgical Time (Minutes)	149.0 [95.0 to 237.0]	185.5 [107.0 to 288.0]	< 0.001
Insulin Given	239 (6.6)	788 (55.7)	< 0.001
**Outcomes**			
30 Day Morbidity and Mortality^a^	1109 (30.9)	538 (38.1)	< 0.001
Infectious Complications	622 (17.3)	287 (20.3)	0.013

Data are presented as frequency (%) or median [25th percentile to 75th percentile], as appropriate

^a^Percentages for diabetic/non-diabetic are given as percent of the non-missing number of values in that group

There was no significant collinearity between the model variables, so all were included. After adjusting for the model covariates, there was no statistically significant difference in the odds of 30-day combined morbidity and mortality between those with a glucose > 180 mg/dL compared to those with a glucose ≤180 mg/dL (adjusted OR 1.1, 95% CI: 0.9, 1.3; *p* = 0.623; Table 3). This model had a c-statistic of 0.720. The same was true for the outcome of infectious complications (adjusted OR 0.90, 95% CI: 0.70, 1.2; *p* = 0.502; model c-statistic of 0.709; Table 3). A subgroup analysis of only those without diabetes revealed the same absence of statistical significance for both the primary outcome (AOR 0.9, 95% CI: 0.6, 1.3; *p* = 0.544) and secondary outcome (AOR 0.8, 95% CI: 0.5, 1.2; *p* = 0.207). Similar results were found for both outcomes in the subgroup analyses for elective cases, admit status cases, inpatient status cases, diabetic only cases, non-diabetic only cases, and surgery duration longer than 60 min. Finally, the sensitivity analysis for a glucose > 150 mg/dL confirmed the absence of statistically or clinically significant relationship with the primary outcome (AOR 1.1, 95% CI: 0.9, 1.3; *p* = 0.287) and secondary outcome (AOR 1.0, 95% CI: 0.8, 1.2; *p* = 0.997). Results from our post hoc sensitivity analysis revealed a small statistically significant increase in the odds of 30-day postop morbidity and mortality for every 20 mg/dl increase in maximum blood glucose over 180 (AOR 1.08, 95% CI: 1.04, 1.12; *p* < 0.001) after adjusting for the other specified model covariates. There was no statistically significant increase in the odds of infectious complications for every 20 mg/dl increase in maximum blood glucose over 180 (AOR 1.01, 95% CI: 0.97, 1.06; *p* = 0.646) after adjusting for the other specified model covariates. In the post-hoc sensitivity analysis evaluating a hyperglycemia threshold of blood glucose ≥250 mg/dL, these patients had 1.59 times the odds of having 30-day morbidity and mortality than those with a peak intraoperative blood glucose < 250 mg/dL (adjusted odds ratio: 1.59, 95% CI: 1.24, 2.05; *p* < 0.001), but did not have a statistically significantly higher odds of 30-day infectious complications (adjusted odds ratio: 1.14, 95% CI: 0.85, 1.52; *p* = 0.386).

**Table 3:** Adjusted Primary and Secondary Outcomes

	Primary Outcome: 30-Day Combined Morbidity and Mortality			Secondary Outcome: 30-Day Infectious Complications		
	Adjusted Odds Ratio	95% Confidence Interval	*P*-Value	Adjusted Odds Ratio	95% Confidence Interval	*P*-Value
Age	**1.01**	**1.01, 1.02**	**< 0.001**	1.01	1.00, 1.01	0.193
Female Sex	0.99	0.85, 1.16	0.903	1.01	0.85, 1.20	0.908
*Race*						
White (ref)						
American Indian or Alaska Native	0.48	0.10, 2.25	0.352	0.77	0.17, 3.57	0.736
Asian	**2.38**	**1.09, 5.22**	**0.030**	1.59	0.65, 3.87	0.308
Black or African American	**0.76**	**0.61, 0.94**	**0.013**	0.87	0.68, 1.11	0.253
Native Hawaiian or Pacific Islander	3.04	0.07, 130.47	0.563	4.70	0.14, 154.14	0.385
Unknown	0.84	0.56, 1.27	0.402	0.93	0.59, 1.47	0.761
*WHO BMI Classification*						
Normal (ref)						
Underweight	1.16	0.68, 1.27	0.589	**1.79**	**1.04, 3.10**	**0.036**
Overweight	0.96	0.77, 1.67	0.675	1.08	0.85, 1.39	0.525
Obese	0.98	0.80, 1.19	0.868	1.04	0.82, 1.31	0.764
*ASA Class*						
2 (ref)						
1	< 0.001	< 0.001, > 999	0.972	< 0.001	< 0.001, > 999	0.977
3	**1.44**	**1.08, 1.91**	**0.012**	1.29	0.95, 1.77	0.108
4	**2.41**	**1.75, 3.33**	**< 0.001**	**1.63**	**1.13, 2.33**	**0.008**
*Procedure Category*						
General (ref)						
Endocrine	1.38	0.98, 1.93	0.066	1.38	0.95, 2.01	0.091
GI (Upper or Colorectal)	**1.94**	**1.53, 2.46**	**< 0.001**	**1.85**	**1.41, 2.41**	**< 0.001**
Hepatobiliary	**0.67**	**0.48, 0.93**	**0.017**	0.85	0.59, 1.23	0.394
Vascular	**0.62**	**0.48, 0.81**	**< 0.001**	**0.52**	**0.39, 0.70**	**< 0.001**
Urgent/Emergent Case	**1.90**	**1.61, 2.25**	**< 0.001**	**1.50**	**1.24, 1.81**	**< 0.001**
Year 2013	1.19	0.98, 1.45	0.080	**1.32**	**1.06, 1.66**	**0.015**
Year 2014	1.02	0.84, 1.23	0.869	1.15	0.92, 1.43	0.230
Intraop Insulin Administered	1.20	0.96, 1.48	0.104	**1.31**	**1.03, 1.67**	**0.027**
Surgical Time (per minute)	**1.004**	**1.003, 1.004**	**< 0.001**	**1.003**	**1.002, 1.003**	**< 0.001**
Number of Comorbidities^a^	**1.16**	**1.11, 1.22**	**< 0.001**	**1.21**	**1.15, 1.28**	**< 0.001**
Blood Glucose > 180 mg/dl	1.05	0.86, 1.28	0.623	0.93	0.74, 1.16	0.502

^a^Comorbidities include diabetes, ventilator dependence, COPD, pneumonia, ascites, congestive heart failure, hypertension, history of peripheral vascular disease, currently requiring or on dialysis, disseminated cancer, open wound, use of steroids/immunosuppressive medications for chronic condition, > 10% loss of body weight in the 6 months prior to surgery, alcohol use > 2 drinks/day in the 2 weeks prior to surgery, presence of sleep apnea, cigarette use within 1 year, presence of sepsis or severe sepsis within 48 h prior to surgery, history of coronary artery disease, and history of deep vein thrombosis

## Discussion

The results from this study of surgical registry patients with glucose measurements performed intraoperatively demonstrate no statistically significant difference between patients who had intraoperative glucose > 180 mg/ dL versus those ≤180 mg/ dL regardless of diabetes status. There are no published multicenter data evaluating intraoperative glucose data across a large and generalizable population. The SCOAP-CERTAIN study demonstrated that hyperglycemic patients without diabetes had higher rates of complications than patients with diabetes. This study had a similar patient population but could not evaluate intraoperative glucose data [10].

The World Health Organization (WHO) has published guidelines regarding surgical site infection (SSI) reduction, including recommending intensive glycemic control in the perioperative period for diabetes and non-diabetes patients, although the level of evidence is of low quality [22]. These guidelines have led to initiatives incorporating glycemic control including targeting an intraoperative value of 180 mg/dl [23]. Two landmark trials that have shaped practice both studied interventions in critical care units. The Leuven trial concluded that tight glucose control (glucose at or below 110 mg/dL or 6.1 mmol/L) significantly reduced morbidity and mortality in critically ill patients, while NICE SUGAR Study found that intensive glucose control (81 to 108 mg/dL or 4.5 to 6 mmol/L) increased mortality compared to a liberal target (less than 180 mg/dL) [20, 24]. Neither one of these trials included intraoperative data. Overall, there is limited evidence for treatment thresholds in the intraoperative period. A recent meta-analysis demonstrated reduced postoperative mortality with moderate (between 150 and 200 mg/dL) vs liberal (greater than 200 mg/dL) targets, but no difference in outcome between moderate vs strict control (less than 150 mg/dL). However, this analysis was not specific to the intraoperative period [2].

The current data questions the scientific basis of intensive or tight glucose intraoperative protocol for non-cardiac cases. Our sensitivity analysis demonstrated no statistically significant difference in outcomes between glucose less than or greater than 150 mg/dL or 8.3 mmol/L. This corroborates findings from the meta-analysis, and strengthens the reproducibility and reliability of our observations. Many anesthesiologists are reluctant to administer insulin to non-diabetics with hyperglycemia intraoperatively due to potentially devastating effects of hypoglycemia under anesthesia. Knowing that moderate vs strict control of hyperglycemia may not be harmful can be reassuring to this group. Post hoc sensitivity analysis of blood glucose ≥250 demonstrating higher morbidity/mortality does reinforce that poorly controlled blood glucose may be harmful, but we did not observe this finding for our secondary outcome of infectious complications.

Hyperglycemia in non-diabetic patients, sometimes known as stress induced hyperglycemia (SIH), is associated with poorer outcomes compared to hyperglycemia in patients with diabetes [25]. In these cases, hyperglycemia can be a response to acute illness or injury. Even though glucose returns to normal after the illness or injury abates, hyperglycemia, including pre-admission glycemic control and admission hyperglycemia, appears to be independently associated with perioperative morbidity [26]. Emerging research has demonstrated that there may be additional factors, such as intraoperative glucose variability, that impact postoperative morbidity [27, 28]. These findings underscore the need for additional research into specific treatment thresholds based on patient comorbidities and physiologic response to surgery [29].

Despite the limitations of this study (described below), our findings support the need for a less strict intraoperative glycemic control. Furthermore, there may be significant opportunities for practice improvement in measurement, treatment and monitoring of intraoperative glucose. Patients with fewer comorbidities, but additional risk due to stress hyperglycemia or glucose variability may need more glucose testing than they are currently obtaining. Additional testing may uncover patients with undiagnosed diabetes and prediabetes [25]. Finally, real- time alerting systems can help providers adhere to standard of care practices during the intraoperative period and reduce the incidence of both hyper and hypoglycemia [15].

### Limitations

We found ~ 5000 cases (out of almost 93,000 cases) had intraoperative glucose measurements. This was lower than we expected in this large general surgery population. However, we believe this represents the actual care provided from this broad representation of hospital types since the nurse abstractors assigned to the MSQC are specifically trained to obtain the information required by the registry. As one can imagine, there is wide variation in culture and practice patterns across hospitals to perform intraoperative point-of-care testing. However, given the lack of electronic medical records in some hospitals, there is a possibility of missing data due to manual abstraction from paper records. This dataset did not provide access to the specific time intraoperatively that the peak glucose was recorded, nor did we have access to subsequent glucose values to understand results of treatment.

The data on insulin administration suggests that there are patients with hyperglycemia in the perioperative period that this study does not capture. Among the patients with diabetes (18,207/92,751) insulin was given to 942 patients, but only 824 of these had a documented intraoperative blood glucose. We think it is likely that these remaining 118 patients who received insulin had high blood glucose values, but this was not captured by our study dataset, perhaps because blood glucose values were only measured preoperatively and not intraoperatively. Future studies could look at a MSQC dataset combined with glucose measurements performed before and after the intraoperative time period to obtain a broader assessment of perioperative glucose management.

Finally, this study is limited by the factors that limit all retrospective designs: confounders, inability to assert causality, and selection bias [30].

## Conclusion

Perioperative glycemic management is an important part of anesthetic care. In our study, we found no statistically significant difference in 30-day combined morbidity and mortality or 30-day infectious complications between patients who had peak glucose levels greater than or less than 180 mg/ dL, and conclude that this moderate glycemic target is not associated with poor outcomes in our multicenter sample of general surgery cases.

## Data Availability

The datasets generated during and/or analyzed during the current study are available from the corresponding author on reasonable request. The source of this data is the Michigan Surgical Quality Collaborative registry.

## Abbreviations

ASA: American Society of Anesthesia
BMI: Body Mass Index
CAD: coronary artery disease
COPD: chronic obstructive pulmonary disease.
CI: confidence interval
GI: gastrointestinal
MSQC: Michigan Surgical Quality Collaborative
AOR: adjusted odds ratio
SIH: stress induced hyperglycemia
STROBE: Strengthening the Reporting of Observational Studies in Epidemiology
SSI: Surgical Site Infection
WHO: World Health Organization
